# Flavonoids and Saponins from Two *Chenopodium* Species (*C. foliosum* Asch. and *C. bonus*-*henricus* L.)—Preliminary Evaluation for hMAO-A/B, Neuroprotective Activity, and Validated UHPLC-HRMS Quantification of Ethanolic Extract from *C. foliosum*

**DOI:** 10.3390/molecules30051061

**Published:** 2025-02-26

**Authors:** Magdalena Kondeva-Burdina, Dona Panayotova, Paraskev T. Nedialkov, Zlatina Kokanova-Nedialkova

**Affiliations:** 1 Department of Pharmacology, Pharmacotherapy and Toxicology, Faculty of Pharmacy, Medical University of Sofia, 2 Dunav Str., 1000 Sofia, Bulgaria; mkondeva@pharmfac.mu-sofia.bg (M.K.-B.); 118848@students.mu-sofia.bg (D.P.); 2 Pharmacognosy Department, Faculty of Pharmacy, Medical University of Sofia, 2 Dunav Str., 1000 Sofia, Bulgaria

**Keywords:** flavonoids, saponins, *Chenopodium foliosum*, *Chenopodium bonus*-*henricus*, MAOA/B, neurotoxicity, neuroprotection, GSH, ROS, UHPLC-HRMS quantification

## Abstract

The development of more effective treatments for neurodegenerative disorders presents a significant challenge in modern medicine. Currently, scientists are focusing on discovering bioactive compounds from plant sources to prevent and treat neurodegenerative diseases. Fifteen flavonoids and saponins from *C. foliosum* Asch. and *C. bonus*-*henricus* L. were tested for their inhibitory activity on hMAO-A and hMAO-B. Five compounds (1 μM) exhibit a weak inhibitory effect on hMAO-A and show good inhibitory activity against the hMAO-B enzyme (30–35%), compared to the positive control selegiline (55%). These active compounds were examined on rat brain synaptosomes and mitochondria obtained by multiple differential centrifugations using a Percoll gradient. Their effects were also monitored on rat brain microsomes obtained by double differential centrifugation. The main parameters characterizing the functional–metabolic status of subcellular fractions are synaptosomal viability, GSH level, and MDA production. All tested compounds (50 μM) demonstrated significant neuroprotective and antioxidant activities across models of induced oxidative stress, including 6-OHDA, t-BuOOH, and Fe^2+/^AA-induced lipid peroxidation. The plausible mechanisms of neuroprotection rely on MAO-B inhibition, the scavenging of ROS, stabilizing the cell membrane by reducing MDA production, and neutralizing free radicals by maintaining GSH levels. In addition, we developed and validated a UHPLC-HRMS method for identifying and simultaneously quantificatying flavonoids and saponins in the aerial parts of *C. foliosum*. Compounds 30-normedicagenic acid- HexA-Hex-TA **22f** and medicagenic acid-HexA-Hex-TA **25f** were considered new natural compounds.

## 1. Introduction

The genus *Chenopodium* (Amaranthaceae) includes over 200 species and is found on all continents except Antarctica and certain archipelagos like New Zealand, Hawaii, and Juan Fernandez [1]. *Chenopodium bonus*-*henricus* L. (family Amaranthaceae) is commonly found across Europe, Western Asia, and North America. In several European countries, the leaves of this plant are used as a spinach substitute. At the same time, shoots and flower clusters are consumed similarly to asparagus and broccoli [2]. In Bulgarian folk medicine, the roots of *C. bonus*-*henricus* are used to treat various ailments, including bronchitis, laryngitis, rheumatism, gout, constipation, and dermatitis. A decoction made from the roots is also utilized in the food industry for producing “tahin” and “white halva” [3].

Nine flavonoids, specifically glycosides of patuletin, 6-methoxykaempferol, and spinacetin, were isolated from the aerial parts of *C. bonus*-*henricus* [2]. Additionally, six saponins, including phytolaccagenin, 2β-hydroxyoleanoic acid, bayogenin, 2β-hydroxygypsogenin, and medicagenic acid, were extracted from the roots of the same plant [3]. Isolated compounds have demonstrated hepatoprotective and antioxidant activities comparable to those of silibinin/silymarin in in vitro models of carbon tetrachloride (CCl_4_)-induced liver damage [1,2]. Additionally, these compounds have shown significant neuroprotective effects on isolated rat brain synaptosomes using a 6-hydroxydopamine in vitro model [1,4] and in a model of hydrogen peroxide (H_2_O_2_)-induced oxidative stress on human neuroblastoma SH-SY5Y cells [5]. Furthermore, isolated flavonoids and saponins exhibited anti-α-glucosidase and prolipase activities [1,4,6]. The flavonoids exhibited DPPH and ABTS radical-scavenging activity and significantly inhibited lipid peroxidation in a linoleic acid system, determined by the ferric thiocyanate method [1]. Furthermore, the saponins tested exhibited moderate to marginal cytotoxicity on five leukemic cell lines (HL-60, SKW-3, Jurkat E6-1, BV-173, and K-562) and enhanced interleukin-2 production in PHA/PMA-stimulated Jurkat E6-1 cells [3].

*Chenopodium foliosum* Asch. (Amaranthaceae) is an annual herb distributed throughout Europe, North Africa, and Central and Southwest Asia [7]. In Bulgarian folk medicine, the plant has also been known as “garliche” or “svinski yagodi” (swine’s berries), and the decoction of its aerial parts has been used for the treatment of cancer and as an immunostimulant and antioxidant drug [8]. A phytochemical study of the aerial parts of the plant has resulted in the isolation of six flavonol glycosides, including patuletin, 6-methoxykaempferol, spinacetin, and gomphrenol [8,9]. Additionally, four glycosides of 30-normedicagenic acid with stimulatory effects on interleukin-2 production were identified [10]. Some of these flavonoids have exerted in vitro hepatoprotective activity in a model of CCl_4_-induced liver damage and have shown antioxidant activity [1,9].

Monoamine oxidase (MAO) is a flavin-adenine-dinucleotide-dependent enzyme located on the outer membrane of mitochondria. There are two isoforms of MAO: MAO-A and MAO-B [11]. Both MAO-A and MAO-B play a role in regulating amine neurotransmitters, including dopamine (DA). MAO-A is responsible for metabolizing DA in presynaptic neurons, while MAO-B metabolizes DA, which is released into the synaptic cleft and subsequently taken up by glial cells. The number of glial cells tends to increase with age and in neurodegenerative diseases, and MAO-B activity also rises under these conditions [12]. Inhibitors of monoamine oxidase A (MAO-A) and monoamine oxidase B (MAO-B) are clinically used to treat psychiatric and neurodegenerative diseases, respectively. MAO-B inhibitors increase the levels of dopamine in the brains of individuals with Parkinson’s disease, especially in cases where there is a partial depletion of dopamine-producing neurons in the substantia nigra pars compacta (SNpc), thus providing an anti-Parkinsonian effect [13]. On the other hand, MAO-A inhibitors are primarily used as antidepressants [14].

Parkinson’s disease is the second most common neurodegenerative disease, following Alzheimer’s. It is predicted that by 2040, the number of diagnosed patients will reach 12 million [15,16]. Parkinson’s disease is characterized by the abnormal formation of Lewy neurites and Lewy bodies, consisting of intraneuronal protein aggregates, primarily α-synuclein. These aggregates spread ascending from the medulla oblongata to the pontine tegmentum, midbrain, mesocortex, and neocortex. Additionally, there is a decrease in the number of dopaminergic neurons in the substantia nigra, which leads to a deficiency of dopamine [17,18,19]. The most common motor symptoms are bradykinesia, resting tremor, rigidity, postural instability, and gait disorder. These symptoms are often accompanied by various non-motor symptoms, including sleep disturbances, depression, and fatigue [20]. In Parkinson’s disease, the overproduction of reactive oxygen species (ROS) is exacerbated by factors such as neuroinflammation, mitochondrial dysfunction, aging, and elevated levels of iron and calcium, as well as the degradation of dopamine [21]. The levels of malondialdehyde, a marker of the lipid peroxidation process, are also increased [22].

**Figure 1:**
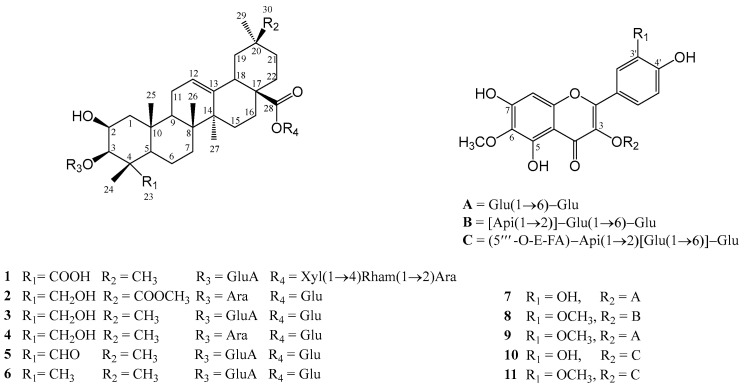
2D ChemBioDraw Ultra 13.0 structures of saponins and flavonoids from *C. bonus*-*henricus* L. (roots and aerial parts).

**Figure 2:**
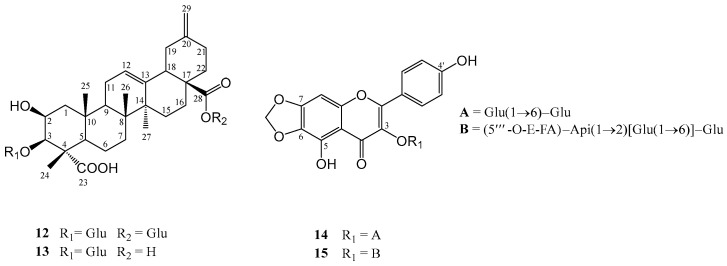
2D ChemBioDraw Ultra 13.0 structures of saponins and flavonoids from the aerial parts of *C. foliosum* Asch.

In this paper, we continue our research on the pharmacology and phytochemistry of two Bulgarian species by investigating 15 flavonoids and saponins from *C. foliosum* and *C. bonus*-*henricus* for their inhibitory activity against hMAO-A/B. We examined the active compounds in various subcellular fractions of the rat brain, including synaptosomes, mitochondria, and microsomes. Additionally, we developed and validated a UHPLC-HRMS method for the simultaneous quantification of flavonoids and saponins present in the aerial parts of *C. foliosum*. The structures of the tested compounds are illustrated in Figure 1 and Figure 2.

## 2. Results and Discussion

### 2.1. Effects of the Compounds ***1***–***15*** on Human Recombinant MAO-A/MAO-B Enzyme

**Figure 3:**
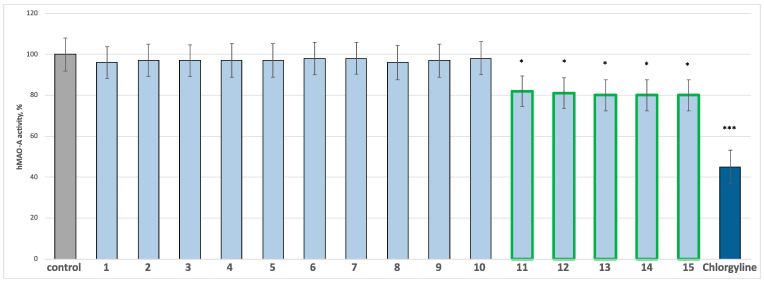
Effects of the compounds **1**–**15** and the positive control chlorgyline (administered alone at 1 µM concentration) on human recombinant MAOA enzyme (hMAOA) activity. * *p* < 0.05; *** *p* < 0.001 vs. control (pure hMAOA). The green color indicates the active compounds.

**Figure 4:**
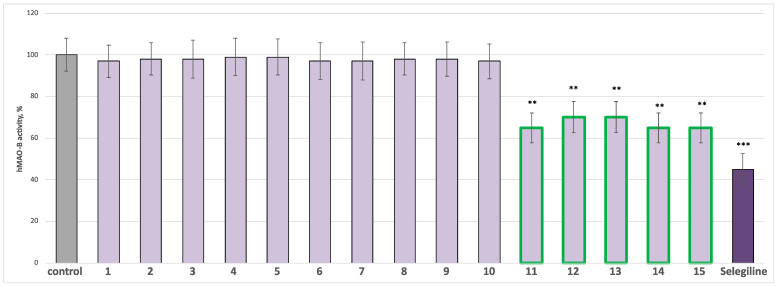
Effects of the compounds **1**–**15** and the positive control selegiline (administered alone at 1 µM concentration) on the activity of the human recombinant MAOB enzyme (hMAOB). ** *p* < 0.01; *** *p* < 0.001 vs. control (pure hMAOB). The green color indicates the active compounds.

According to the published protocols, saponins and flavonoids from *C. bonus*-*henricus* and *C. foliosum* were screened for potential MAOA and MAOB inhibitory activity [23]. Chlorgyline and selegiline were positive controls in hMAO-A and hMAO-B tests, respectively. All the substances and positive controls were applied at a final concentration of 1 µM. The results are presented in Figure 3 and Figure 4. When administered alone at a tested concentration, compounds **1**–**10** showed no statistically significant MAO-A/B inhibitory effects compared to the control. Only spinacetin triglycoside **11,** as well as 30-normedicagenic acid glycosides **12**–**13** and gomphrenol glycosides **14**–**15,** exhibited a weak, statistically significant MAO-A and good MAO-B inhibitory effect compared to the controls (pure hMAO-A and pure hMAO-B). Compound **11** inhibited hMAO-A by 18%, compound **12** by 19%, and compounds **13**–**15** by 20%. The classical MAO-A inhibitor chlorgyline significantly reduced the enzyme activity by 55% compared to the control (pure hMAOA) (Figure 3). Furthermore, flavonoids **11**, **14** and **15** reduced the hMAO-B activity by 35%, while saponins **12** and **13** reduced it by 30%. Selegiline, the classic MAO-B inhibitor, reduced the enzyme activity by 55% (Figure 4). Additionally, we examined the active compounds for neuroprotective effects in various subcellular fractions of the rat brain, including synaptosomes, mitochondria, and microsomes.

### 2.2. Neuroprotective Effects of the Compounds ***11***–***15*** on Isolated Rat Brain Synaptosomes, Mitochondria, and Microsomes

#### 2.2.1. Effects of the Compounds **11**–**15** on Isolated Rat Brain Synaptosomes

**Figure 5:**
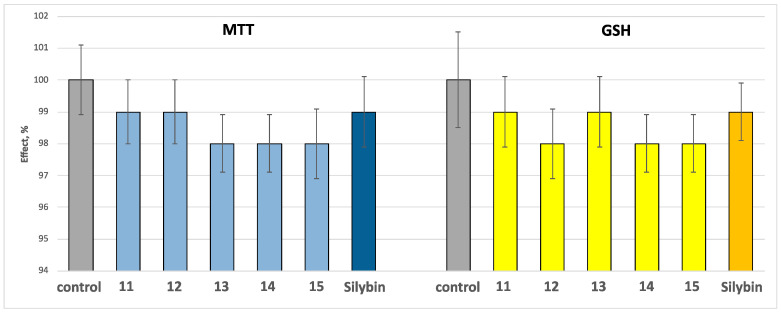
Toxic effects of the tested compounds **11**–**15**, and the positive control silybin, administered alone at a concentration of 100 μM on synaptosomes viability and GSH level at isolated rat brain synaptosomes.

The neurotoxicity of the compounds **11**–**15** and the positive control silybin on biomarkers, characterizing the functional–metabolic profile of brain synaptosomes, was investigated. When administered alone, at a relatively high concentration (100 µM), compounds **11**–**15** did not exhibit a statistically significant neurotoxic effect on isolated rat brain synaptosomes. They do not statistically significantly change the main parameters characterizing the functional–metabolic status of synaptosomes—the synaptosomal vitality determined by MTT-test and the level of reduced glutathione (GSH) (Figure 5).

Treatment of synaptosomes with 6-hydroxydopamine (6-OHDA) is a known in vitro model that mimics some of the symptoms of Parkinson’s disease. 6-OHDA has some structural similarities to dopamine (DA) and norepinephrine (NA) and exhibits a high affinity for several catecholaminergic transporters in the plasma membrane, including the dopamine (DAT) and noradrenaline (NET) transporters. Therefore, the compound can enter dopaminergic and noradrenergic neurons and damage the peripheral and central nervous systems. Once inside the neuron, 6-OHDA is stored in granulation structures and can be released upon nerve stimulation, thus acting as a neurotransmitter. MAO (monoamine oxidase) is an important enzyme for the metabolism of 6-OHDA. This enzyme, as well as storage in granulation structures, may serve as protective mechanisms [24]. At high concentrations in the cytoplasm, 6-OHDA generates highly reactive products—peroxides, superoxides, and quinones. These products react nonspecifically with neuronal structures and damage neurons [24].

**Figure 6:**
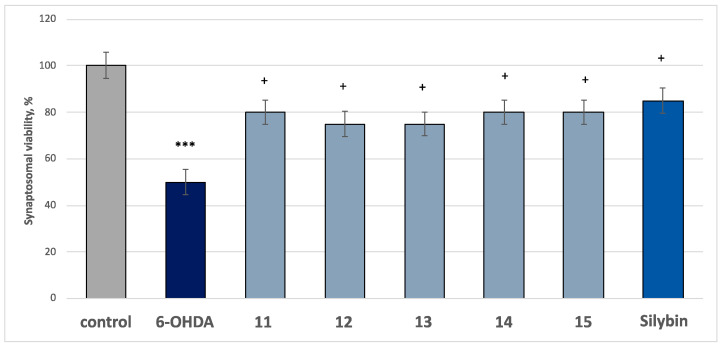
Effects of the tested compounds **11**–**15** (50 μM) and the positive control silybin (50 μM) in combination with 6-OHDA (150 μM) on synaptosomes viability at isolated rat brain synaptosomes. *** *p* < 0.001 vs. control (non-treated synaptosomes); ^+^*p* < 0.05 vs. control (pure 6-OHDA).

**Figure 7:**
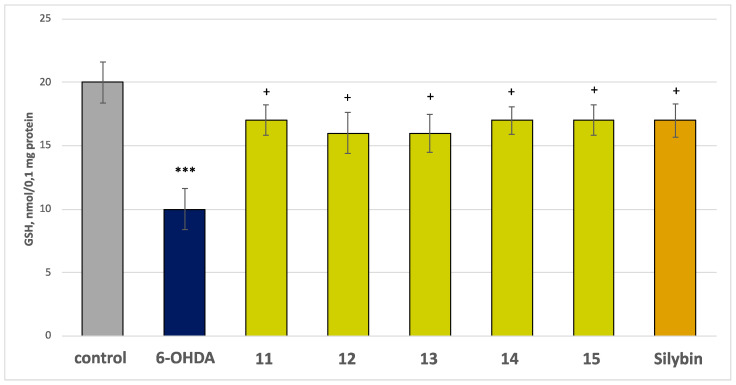
Effects of the tested compounds **11**–**15** (50 μM) and the positive control silybin (50 μM) in combination with 6-OHDA (150 μM) on GSH level at isolated rat brain synaptosomes. *** *p* < 0.001 vs. control (non-treated synaptosomes); ^+^*p* < 0.05 vs. control (pure 6-OHDA).

In this model of neurotoxicity, the tested compounds **11**–**15**, at a concentration of 50 µM, exhibited good statistically significant neuroprotective and antioxidant activity compared to the control (pure 6-OHDA). Applied alone, at a concentration of 150 µM, the toxic agent (6-OHDA) significantly reduced synaptosomal viability and GSH level by 50% compared to the control (untreated synaptosomes) (Figure 6 and Figure 7).

In combination with 6-OHDA, all the compounds, and silybin, at a concentration of 50 µM, preserved synaptosome vitality and GSH levels as follows: compounds **11**, **14**, and **15** preserved synaptosome viability by 60%, compounds **12** and **13**—by 50%, and silybin—by 70%, compared to the control (pure 6-OHDA) (Figure 6); flavonoids **11**, **14**, **15** and silybin preserved the GSH level by 70%, while saponins **12** and **13** preserved this parameter by 60%, compared to the control (pure 6-OHDA) (Figure 7). The results showed that the most active were the glycosides of spinacetin and gomphrenol, which was probably due to the presence of esterified ferulic acid in their moiety, which is well known for its antioxidant properties and ability to scavenge ROS.

The possible mechanism of neuroprotective activity in this model of neurotoxicity is most likely related to the inhibition of MAO-B activity on the one hand and preservation of the level of reduced glutathione, the main nucleophile that captures free radicals on the other hand.

#### 2.2.2. Effects of the Compounds **11**–**15** on Isolated Rat Brain Mitochondria

**Figure 8:**
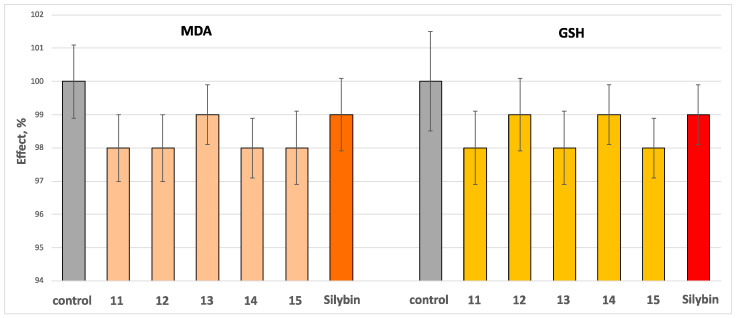
Effects of the tested compounds **11**–**15**, and the positive control silybin, administered alone at a concentration of 100 μM on MDA production and GSH level at isolated rat brain mitochondria.

When administered individually at a concentration of 100 μM, compounds **11**–**15** and silybin did not show statistically significant neurotoxic effects on isolated rat brain mitochondria. Additionally, these compounds did not produce significant changes in the key biomarkers that indicate the functional and metabolic status of mitochondria, namely the production of malondialdehyde (MDA) and the level of reduced glutathione (GSH) (Figure 8).

Oxidative stress is one of the main mechanisms by which neurotoxic compounds induce apoptosis or necrosis [25]. A widely used experimentally in vitro model for inducing oxidative stress is with the toxic agent that-butyl hydroperoxide (t-BuOOH). Several mechanisms of action for t-BuOOH have been described, including the formation of free radicals, a decrease in glutathione reductase activity, and an increase in glutathione peroxidase activity, which contributes to a reduction in GSH levels; it also oxidizes the sulfhydryl (-SH) groups of mitochondrial enzymes and inhibits cellular respiration [26].

**Figure 9:**
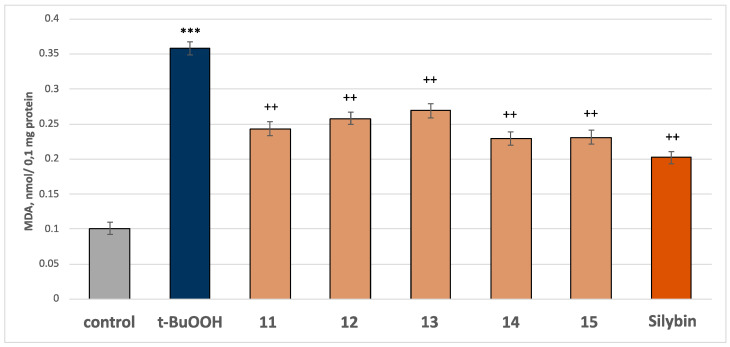
Effects of the tested compounds **11**–**15** and the positive control silybin, at a concentration of 50µM, in combination with t-BuOOH (75 µM) on MDA production at isolated rat brain mitochondria. *** *p* < 0.001 vs. control (non-treated mitochondria); ^++^ *p* < 0.01 vs. control (pure t-BuOOH).

**Figure 10:**
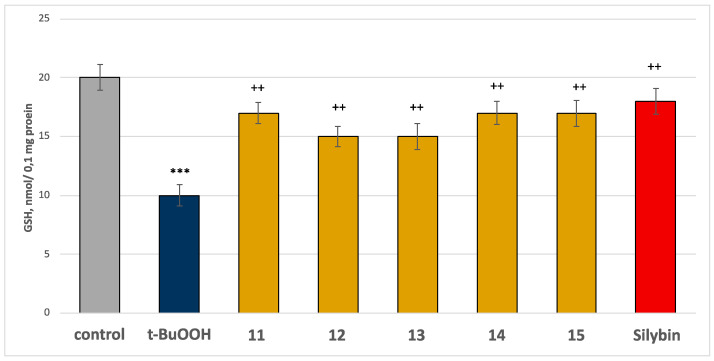
Effects of the tested compounds **11**–**15** and the positive control silybin, at a concentration of 50 µM, in combination with t-BuOOH (75 µM) on GSH level at isolated rat brain mitochondria. *** *p* < 0.001 vs. control (non-treated mitochondria); ^++^ *p* < 0.01 vs. control (pure t-BuOOH).

In this model of neurotoxicity, all the tested compounds at a concentration of 50 µM exhibited good neuroprotective and antioxidant activity compared to the control (pure t-BuOOH). When administered alone at a concentration of 75 µM, t-BuOOH significantly increased MDA production by 254% and reduced GSH levels by 50% compared to the control group (non-treated mitochondria) (Figure 9 and Figure 10).

In combination with t-BuOOH, all the compounds, along with silybin, at a concentration of 50 µM, preserved the GSH level and reduced MDA production. Specifically, compound 11 reduced MDA production by 32%, compound 12 by 28%, compound 13 by 25%, compound 14 by 36%, compound 15 by 35%, and silybin by 44%, compared to the control (pure t-BuOOH) (Figure 9). Regarding GSH levels, compounds **11**, **14**, and **15** preserved the GSH level by 70%, compounds **12** and **13**—by 50%, and silybin—by 80%, compared to the control (pure t-BuOOH) (Figure 10). The results showed that flavonoids preserved the GSH level and reduced MDA production better than the tested saponins. The most active flavonoids are glycosides of gomphrenol (compounds **14** and **15**).

The established neuroprotective and antioxidant effects of the tested compounds in a model of tert-butyl hydroperoxide-induced oxidative stress are likely related to the following: (1) stabilizing the cell membrane by reducing MDA production, a classical biomarker of lipid peroxidation, and (2) neutralizing free radicals by maintaining GSH levels, which is the primary nucleophile responsible for eliminating free radicals and reactive metabolites.

#### 2.2.3. Effects of the Compounds **11**–**15** on Isolated Rat Brain Microsomes

Microsomes are heterogeneous, vesicle-like fragments ranging from 20–200 nm in diameter, formed in vitro from parts of the endoplasmic reticulum during the fragmentation of eukaryotic cells. These structures are not present in healthy, living cells. Microsomes can be concentrated and separated from other cell organelles through repeated centrifugation. Microsomes were used as a lipid membrane model in experiments related to lipid peroxidation [27].

**Figure 11:**
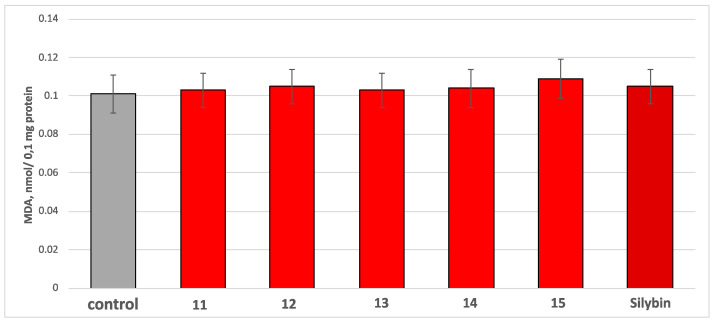
Effects of the tested compounds **11**–**15** and silybin administered alone at 100 µM on MDA production on isolated rat brain microsomes.

The compounds **11**–**15** and silybin applied alone, at a concentration of 100 µM, did not show any pro-oxidant effects on isolated rat brain microsomes. They did not alter malondialdehyde (MDA) production (Figure 11).

Reactive oxygen species (ROS), such as superoxide anion and hydrogen peroxide, are continuously generated during respiration in aerobic organisms. Although ROS in physiological concentrations is necessary for normal cellular functions, their overproduction can be fatal to the cell because the oxidative stress they induce leads to oxidative damage to lipids, proteins, and DNA. The end products of lipid peroxidation are reactive aldehydes, such as 4-hydroxynonenal and malondialdehyde, which are toxic to the cell. Furthermore, reactive aldehydes generated by lipid peroxidation damage proteins, DNA, and other macromolecules [25].

**Figure 12:**
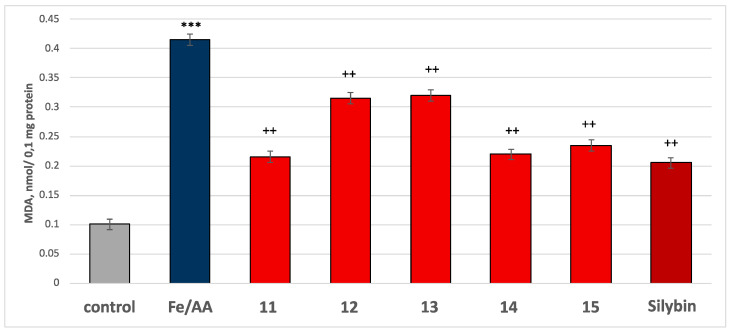
Effects of tested compounds **11**–**15** and silybin, at a concentration of 50 µM, under non-enzyme-induced lipid peroxidation conditions with ferrous sulfate (20 µM)/ascorbic acid (0.5 mM) on MDA production on isolated rat brain microsomes. *** *p* < 0.001 vs. control (non-treated microsomes). ^++^ *p* < 0.01 vs. control (pure iron/ascorbate).

In this model of neurotoxicity, all tested compounds **11**–**15**, at a concentration of 50 µM, demonstrated strong antioxidant activity compared to the control (iron/ascorbic acid). Under conditions of non-enzyme-induced lipid peroxidation, MDA production increased significantly by 311% compared to the control (untreated microsomes) (Figure 12). In this model, all the compounds and silybin, at a concentration of 50 µM, reduced MDA production as follows: compound **11** reduced MDA production by 48%; compound **12** by 24%; compound **13** by 23%; compound **14** by 47%; compound **15** by 43%; and silybin by 51% compared to the control (pure iron/ascorbic acid) (Figure 12). The results indicated that the most active compounds were the glycosides of spinacetin and gomphrenol (compounds **11**, **14**, and **15**).

Based on the experiments conducted, it can be assumed that the mechanism underlying the tested compounds’ antioxidant activity in non-enzyme-induced lipid peroxidation conditions is related to stabilizing the cell membrane by reducing MDA production.

### 2.3. Validation of a UHPLC-HRMS Method for Simultaneously Quantifying Flavonoids and Saponins Content in the Aerial Parts of C. foliosum *Asch.*

#### 2.3.1. Method Validation

**Figure 13:**
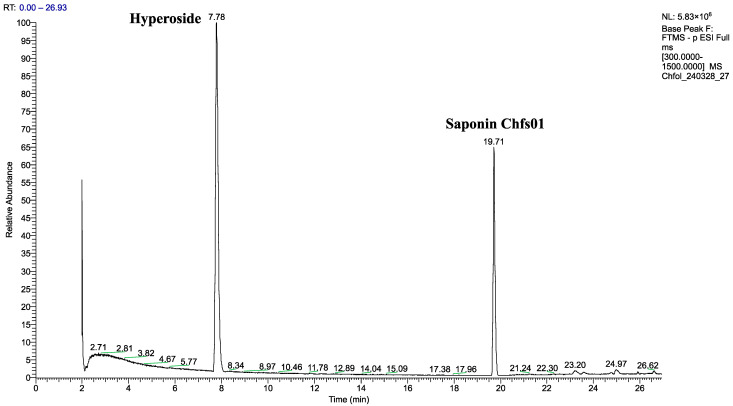
UHPLC-HRMS chromatographic separation of the standards hyperoside and saponin **Chfs01** under the optimized conditions.

This study used UHPLC-HRMS (ultra-high-performance liquid chromatography/high-resolution mass spectrometry) to detect the flavonoids and saponins in the EtOH extract from the aerial parts of *Chenopodium foliosum* Asch. The efficiency of the extraction procedure and optimization of the chromatographic conditions were as given in the literature [28]. Briefly, MeOH, EtOH, and i-PrOH, as well as their mixtures with water, were used as solvents. The best results were obtained with 70% EtOH. Two chromatographic columns, namely Kromasil C18 column (Bohus, Sweden, 2.1 × 100 mm, 1.8 μm) and Kromasil Eternity XT C18 column (2.1 × 100 mm, 1.8 μm) were tested for the separation of flavonoids and saponins from the titled plant. The first column showed the best results and was chosen for method development. The quantitative determination of flavonoids and saponins in the aerial parts of *Chenopodium foliosum* Asch. was performed using the external standard method. Hyperoside and saponin **Chfs01** (3-*O*-β-glucopyranosyl-2β,3β-dihydroxy-30-noroleane-12,20(29)-diene-23,28-dioic acid 28-*O*-β-glucopyranosyl ester) were selected as standards for the calculation of the quantity of detected flavonoids and saponins, respectively. The separation of the standard is given in Figure 13.

The linearity of an analytical method refers to its ability to produce test results that are directly proportional to the concentration of the analyte in the sample within a specific range. Linearity studies are essential because they establish the range of the method where accurate and precise results can be obtained. An analytical method’s limit of detection (LOD) is the smallest amount of analyte in a sample that can be detected, although it may not be quantified with precision. The limit of quantification (LOQ) is the lowest concentration at which the analyte can be quantified with acceptable precision. Both LOD and LOQ are critical parameters in method validation. They provide insights into the sensitivity and reliability of the analytical method, assisting in determining whether it is suitable for detecting and quantifying analytes at low concentrations [29,30]. The calibration curves were linear over the concentration range of 16.41–525 ng/mL and 17.97–575 ng/mL for hyperoside and saponin **Chfs01**, respectively. All the calibration curves showed good linear regressions, and the correlation coefficients were R^2^ > 0.999 (Table 1). The method showed that LODs and LOQs were 0.28 ng/mL and 0.86 ng/mL for hyperoside and 0.48 ng/mL and 1.44 ng/mL for saponin **Chfs01**, respectively (Table 1).

In developing analytical methods, accuracy refers to how closely the actual value aligns with the accepted conventional true value. The importance of accuracy can be described using the term “trueness”. The accuracy of analytes was assessed by conducting the complete extraction procedure on a control plant matrix spiked with a standard solution of analytes at three concentrations similar to those expected in actual plant samples. This approach allows for the evaluation of how effectively the analytical method measures the exact values of the analytes in actual samples [29,30]. The accuracy of the method was checked by the addition of a standard solution mixture at three concentrations (50.20, 100.40, and 150.60 ng/mL for hyperoside; 50.80, 101.60, and 152.40 ng/mL for saponin **Chfs01**) close to that expected in the real plant samples. Blank samples from the same un-spiked plant extract were analyzed at the same time as the spiked samples, and the measured values were subtracted. Furthermore, the related compounds showed overall recoveries ranging from 98.46% to 102.05%, with RSD ranging from 0.38% to 1.22%. The good correlation between spiked (added) and determined concentrations proves that the method has an acceptable accuracy (Table 2).

The precision of an analytical method refers to the closeness of agreement among multiple measurements of the same sample obtained under specific conditions. Intra-day precision (or repeatability) reflects the level of precision achieved under identical operating conditions over a specified short duration (usually ≤1 day). Inter-day precision (or intermediate precision) represents the level of precision obtained while maintaining the same operating conditions, typically within the same laboratory, over a defined period (generally ≥1 day). The precision ensures that measurements taken multiple times yield consistent results, enhancing the data’s reliability. This is essential for drawing accurate conclusions from the analysis [29,31]. The precision of the retention times was established by analyzing the repeated runs during a single day and on three different days, respectively. The RSDs of retention times of the standards were ≤0.07% for intra-day and ≤0.01% for inter-day evaluations, respectively (Table 3). For intra-day and inter-day precision tests, the evaluated analytes exhibited overall recoveries ranging from 97.11% to 98.94%, with RSDs from 0.18% to 0.64% (Table 3).

The developed UHPLC-HRMS method was applied to quantify the flavonoids and saponins detected in the EtOH extract from the aerial parts of *Chenopodium foliosum* Asch.

#### 2.3.2. Detection, Identification, and Quantification of Flavonoids and Saponins in *Chenopodium foliosum*

This work used ultra-high-performance liquid chromatography/high-resolution mass spectrometry (UHPLC-HRMS) to detect and quantify flavonoids and saponins in the aerial parts of *C. foliosum*.

**Figure 14:**
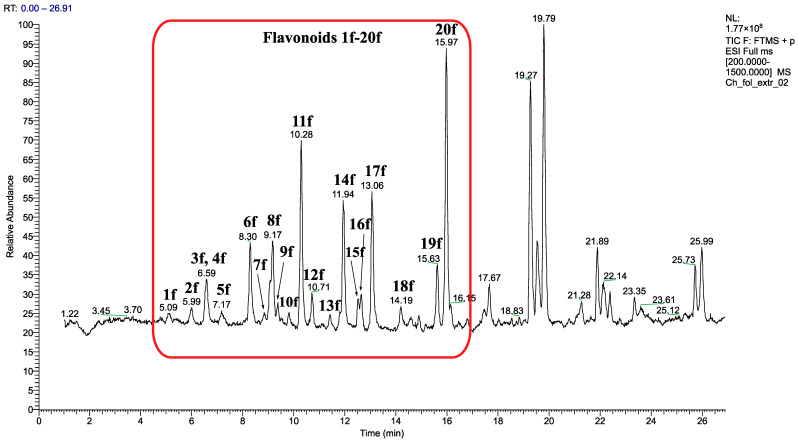
UHPLC-HRMS-based flavonoid profiling of EtOH extract from the aerial parts of *C. foliosum* Asch. in the positive ion mode. The red frame indicates the flavonoids in the chromatogram.

**Figure 15:**
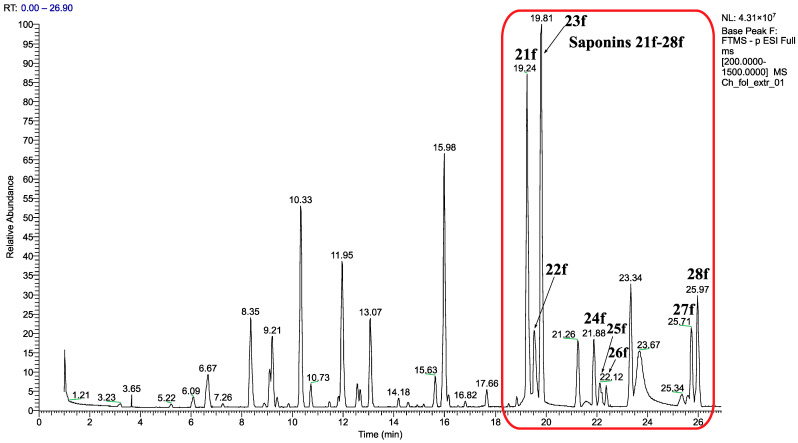
UHPLC-HRMS-based saponin profiling of EtOH extract from the aerial parts of *C. foliosum* Asch. in the negative ion mode. The red frame indicates the saponins in the chromatogram.

Table S1 and Table 4 list the identified flavonoids and saponins and their quantities, while Figure 14 and Figure 15 give the chromatograms of the EtOH extract.

Twenty previously identified flavonoid glycosides with seven aglycones (patuletin, gomphrenol, spinacetin, 6-methoxykaempferol, isorhamnetin, 3,5,3′,4′-tetrahydroxy-6,7-methylenedioxyflavone, and 3,5,4′-trihydroxy-3′-methoxy-6,7-methylenedioxyflavone) were detected [32] (Table S1). Flavonoid composition was dominated by di- and triglycosides and acylated flavonoids. The quantitative determination of flavonoid contents in the aerial parts of *C. foliosum* was performed by the external standard method. The amount of 20 detected flavonoids (Figure 14) was calculated relative to external standard hyperoside (Table 4).

The results (Table 4) showed that the glycosides of gomphrenol (**8f**, **11f**, **14f**, and **20f**) were the main components of the flavonoid mixture with content ranging from 345.09 to 909.03 µg/g D.W. (total 2551.08 µg/g D.W.), calculated as hyperoside. The glycosides of 6-methoxykaempferol (**1f**, **3f**, **6f,** and **17f**) (equivalent to 24.02–326.65 µg/g D.W. hyperoside) (total 806.25 µg/g D.W.) and FLB (**9f**, **12f**, **15f,** and **19f**) (equivalent to 40.04–127.45 µg/g D.W. hyperoside) (total 353.45 µg/g D.W.) were found in smaller quantities. The quantity of the spinacetin (**5f**, **7f,** and **16f**), patuletin (**2f** and **13f**) and FLA (**10f** and **18f**) glycosides was very small, ranging from 16.66 to 74.83 µg/g D.W., calculated as hyperoside.

The total amount of flavonoids was 3997.35 µg/g D.W. Gomphrenol glycosides were 63.81% of the flavonoid mixture. The results also showed that the quantity of pharmacologically tested gomphrenol glycosides, compound **14** (equivalent to **14f**) and compound **15** (equivalent to **20f**), in this work, were 574.16 and 909.03 µg/g D.W., respectively, calculated as hyperoside, which were 37.10% of all the detected flavonoids.

The UHPLC-HRMS-based saponin profiling of aerial parts of *C. foliosum* in a negative ion mode tentatively identified eight saponins of two sapogenins (30-normedicagenic acid and medicagenic acid) (Table S1, Figure 15).

According to the MS, MS^2^ data, and by comparison with references, compounds **21f** (t_R-extr_ = 19.24), **23f** (t_R-extr_ = 19.81), and **28f** (t_R-extr_ = 25.97) (Table S1) were identified as 3-*O*-β-GluA-30-normedicagenic acid-28-*O*-β-Glu, 3-*O*-β-Glu-30-normedicagenic acid-28-*O*-β-Glu, 3-*O*-β-Glu-30-normedicagenic acid, respectively, and were previously isolated from the aerial parts of *C. foliosum* [10].

The compound **22f** (t_R-extr_ = 19.53) exhibited a deprotonated molecule [M−H]^−^ at *m/z* 955.3835, supporting the formula for C_45_H_63_O_22_. Its MS^2^ spectrum showed three fragment ions at *m/z* 823.3763, 647.3480, and 485.2900. The former two ions were due to the sequential loss of tartaric acid (132 Da) and hexauronic acid (176 Da). The later fragment ion corresponded to the cleavage of one hexose unit (162 Da) and was characteristic of 30-normedicagenic acid. The acylation of the glycosyl part was also confirmed by the presence of a characteristic tartaloyl fragment at *m/z* 131.0337. Thus, compound **22f** was tentatively identified as 30-normedicagenic acid-HexA-Hex-TA. The compound **27f** (t_R-extr_ = 25.71) showed a deprotonated molecule [M−H]^−^ at *m/z* 661.3244. Its MS^2^ spectrum gave a fragment ion at *m/z* 485.2898 resulting from a neutral loss of 176 Da, indicating the presence of hexauronic acid. Thus, compound **27f** was established as 30-normedicagenic acid-HexA.

Three glycosides with aglycone medicagenic acid (product ion at *m/z* 501.3222) corresponding to chromatographic peaks **24f** (t_R-extr_ = 21.88), **25f** (t_R-extr_ = 22.12), and **26f** (t_R-extr_ = 22.36) were detected (Table S1). The substance **24f** (t_R-extr_ = 21.88) displayed a deprotonated molecule [M−H]^−^ at *m/z* 839.4090. The MS^2^ fragmentation yielded product ions at *m/z* 663.3695 and 501.3247, which resulted from a cleavage of one hexauronic acid (176 Da) and one hexose unit (162 Da). The later product ion at *m/z* 501.3247 was characteristic of medicagenic acid. Thus, compound **24f** was identified as medicagenic acid-HexA-Hex. The substance **26f** (t_R-extr_ = 22.36) showed a deprotonated molecule [M−H]^−^ at *m/z* 825.4304. Its MS^2^ spectrum gave a fragment ion at *m/z* 439.3207 that corresponded to the cleavage of two hexose units (2 × 162 Da), H_2_O (18 Da), and CO_2_ (44 Da). Thus, compound **26f** was established as medicagenic acid-Hex-Hex. The compound **25f** (t_R-extr_ 22.12) showed a deprotonated molecule [M−H]^−^ at *m/z* 971.4156 (supporting the formula for C_46_H_67_O_22_), which is 132 Da more than that of flavonoid **24f**, suggesting the presence of a tartaloyl unit. Its MS^2^ spectrum showed three fragment ions at *m/z* 839.4084, 663.3749, and 501.3250. The former two ions were due to the sequential loss of tartaric acid (132 Da) and hexauronic acid (176 Da). The later fragment corresponded to the cleavage of one hexose unit (162 Da) and confirmed the presence of sapogenin medicagenic acid. The acylation of the glycosyl part was also confirmed by the presence of a characteristic tartaloyl fragment at *m/z* 131.0337. Thus, compound **25f** was tentatively identified as medicagenic acid-HexA-Hex-TA. Compounds 30-normedicagenic acid- HexA-Hex-TA **22f** and medicagenic acid-HexA-Hex-TA **25f** were suggested to be new natural compounds. Additionally, saponins **24f** (Medicagenic acid-HexA-Hex), **26f** (Medicagenic acid-Hex-Hex), and **27f** (30-normedicagenic acid-HexA) were identified for the first time in *C. foliosum*.

The results (Table 4) showed that the saponins of 30-normedicagenic acid (**21f**–**23f**, **27f**, and **28f**) were the main components of the saponin mixture, with content ranging from 396.61 to 1735.11 µg/g D.W. (total 4587.93 µg/g D.W.), calculated as saponin **Chfs01**. The glycosides of medicagenic acid (**24f**–**26f**) (equivalent to 105.46–366.67 µg/g D.W. saponin **Chfs01**) (total 643.66 µg/g D.W.) were found in small quantities. The total amount of saponins was found to be 5231.59 µg/g D.W. Detected saponins of 30-normedicagenic acid were 87.69% of the saponin mixture.

The results also showed that the quantity of pharmacologically tested 30-normedicagenic acid glycosides, compound **12** (equivalent to **23f**) and compound **13** (equivalent to **28f**) in this work were 1735.11 and 551.17 µg/g D.W., respectively, which was 43.70% of all the detected saponins.

## 3. Materials and Methods

### 3.1. Materials, Chemicals, and Reagents

The pharmacologically tested saponins, 3-*O*-β-D-glucuronopyranosyl-medicagenic acid-28-*O*-β-D-xylopyranosyl(1→4)-α-L-rhamnopyranosyl(1→2)-α-L-arabinopyranosyl ester **1**, Bonushenricoside A **2**, 3-*O*-β-D-glucuronopyranosyl-bayogenin-28-*O*-β-D-glucopyranosyl **3**, 3-*O*-α-L-arabinopyranosyl-bayogenin-28-*O*-β-D-glucopyranosyl ester **4**, 3-*O*-β-D-glucuronopyranosyl-2β-hydroxygypsogenin-28-*O*-β-D-glucopyranosyl ester **5**, and Bonushenricoside B **6** were isolated from the roots of *C. bonus*-*henricus* L. [3]. The tested flavonoids, patuletin-3-*O*-gentiobioside **7**, spinacetin-3-*O*-[β-apiofuranosyl (1→2)]-β-glucopyranosyl(1→6)-β-glucopyranoside **8**, spinacetin-3-*O*-gentiobioside **9**, patuletin-3-O-(5‴-O-E-feruloyl)-β-D-apiofuranosyl(1→2)[β-D-glucopyranosyl (1→6)]-β-D-glucopyranoside **10**, and spinacetin-3-*O*-(5‴-O-E-feruloyl)-β-D-apiofuranosyl (1→2)[β-D-glucopyranosyl(1→6)]-β-D-glucopyranoside **11** were previously isolated from the aerial parts of the same plant [2]. The other investigated saponins (3-*O*-β-glucopyranosyl-2β,3β-dihydroxy-30-noroleane-12,20(29)-diene-23,28-dioic acid 28-*O*-β-glucopyranosyl ester **12** and 3-*O*-β-D-glucopyranosyl-2β,3β-dihydroxy-30-noroleane-12,20(29)-diene-23,28-dioic acid **13**) and flavonoids (gomphrenol-3-*O*-β-gentiobioside **14** and gomphrenol-3-*O*-(5′′′-*O*-E-feruloyl)-β-D-apiofuranosyl (1→2)[β-D-glucopyranosyl-(1→6)]-β-D-glucopyranoside **15**) were previously isolated from the aerial parts of *C. foliosum* [8,9,10].

The reagents and buffers for the pharmacological evaluations, including Percoll rea-gent, glucose, Buffer B, 6-hydroxydopamine (6-OHDA), MTT, dimethyl sulfoxide DMSO, DTNB tert-butyl hydroperoxide (t-BuOOH), trichloroacetic acid, Tris buffer containing dithiothreitol, phenylmethylsulfonyl fluoride, ethylendiamine tetraacetic acid (EDTA), potassium chloride (KCl), and glycerol (pH 7.4), ferrous sulfate, ascorbic acid, silybin, chlorgyline, selegiline, and the corresponding assay kits for the evaluation of MAO inhibitory activity were purchased from Sigma–Aldrich (St. Louis, MO, USA).

All the solvents used in the UHPLC-HRMS analysis were of LC/MS grade. External standard hyperoside (≥97%, HPLC) was purchased from Sigma–Aldrich (Taufkirchen, Germany). Saponin 3-*O*-β-glucopyranosyl-2β,3β-dihydroxy-30-noroleane-12,20(29)-diene-23,28-dioic acid 28-*O*-β-glucopyranosyl (Chfs01) (purity ≥ 97%) was previously isolated from the aerial parts of *Chenopodium foliosum* Asch. [10].

### 3.2. Pharmacological Studies

In the pharmacological experiments, all the tested compounds were dissolved in DMSO and used (1) at a concentration of 100 µM when administered alone; (2) at a concentration of 50 µM in neurotoxicity models; and (3) at a concentration of 1 µM in MAOA/B inhibition testing.

#### 3.2.1. Animals

Thirty animals were used in the experiments. These animals were obtained from the Bulgarian Academy of Sciences National Breeding Center, Slivnitsa, Bulgaria. The animals were kept in plexiglass cages under standard conditions, with free access to water and food. They were exposed to a 12 h light/dark regime at 20–25 °C temperatures. Twelve hours before each specific study, the animals’ food was withheld. The experiments used in this study agreed with the European Communities Council Directive 2010/63/EU for animal experiments and Ordinance No. 15, which outlines the minimum requirements for the protection and welfare of experimental animals (SG No. 17, 2006). The experiments with animals were approved by the Bulgarian Food Safety Agency with Permission No. 273, valid until 20 July 2025.

#### 3.2.2. Determination of Human Recombinant MAO-A/B Enzyme Activity

The activity of recombinant human MAO-A (hMAO-A) and MAO-B (hMAO-B) was determined fluorometrically. Tyramine hydrochloride was used as a substrate. The activity was determined by measuring the production of H_2_O_2_ using the Amplex Red (N-acetyl-3,7-dihydroxyphenoxazine) test in the presence of horseradish peroxidase [23]. Working solutions of the test substances, reagents, and hMAO-A or hMAO-B enzymes were prepared in the reaction buffer according to the manufacturer’s instructions. Pure working solutions of the enzymes in reaction buffer, enzyme solutions containing hydrogen peroxide, and pure reaction buffer were used as controls. The test substances and positive controls were applied at a final concentration of 1 μM. The enzymes and the tested compounds or positive controls were placed in a 96-well plate, with eight replicates per substance or positive control. The plate was incubated in the dark at 37 °C for 30 min. Fluorometric measurements were then performed using a Synergy 2 Microplate Reader at two wavelengths: 570 nm and 690 nm.

#### 3.2.3. Preparation, Isolation, and Incubation of Rat Brain Synaptosomes and Mitochondria

To isolate synaptosomes and mitochondria, a subcellular fractionation method involved a Percoll (a colloidal silica solution) gradient, as described by Taupin et al. [33], with minor modifications. Briefly, a brain homogenate (20 mL in each tube) was prepared and centrifuged at 1000× *g* for 5 min at +4 °C. The supernatant was then collected and subjected to a second centrifugation process, which led to synaptosomes and mitochondria being used for further experiments. The supernatants obtained from the two rounds of centrifugation were combined and divided equally into four tubes. The tubes (20 mL supernatant) were then subjected to three more rounds of centrifugation at 10,000× *g* for 20 min each, at a temperature of +4 °C. The supernatants of the final two centrifugations were used to isolate synaptosomes and mitochondria according to the three-step protocol: (1) a 90% stock solution of Percoll was prepared; (2) stock solutions were diluted to 16% and 10%, and 4 mL of each was placed into six test tubes; and (3) 4 mL of 7.5% Percoll was added to the residue obtained after the last centrifugation. The centrifugation was carried out at +4 °C for 20 min at 15,000× *g*, forming three layers. The bottom, middle, and top layers contain mitochondria, lipids, and synaptosomes (at the 16% to 10% Percoll limit). Each layer from the tubes was collected using a glass pipette and transferred into a separate tube. Buffer B + glucose was added to it. The mixtures were centrifuged at 10,000× *g* for 20 min at +4 °C. Thus, the isolation buffer is exchanged with the incubation buffer. After centrifugation, the pellet with the synaptosomes was mixed and made up with buffer B + glucose. Synaptosomes and mitochondria were incubated with the tested compounds (**11**–**15**) at 100 μM and 50 μM concentrations for 1 h.

#### 3.2.4. In Vitro Dopamine Model of Neurotoxicity

This in vitro model closely resembles the neurodegenerative processes that primarily occur in Parkinson’s disease (PD). The metabolism of 6-OHDA results in the creation of reactive quinones (p-quinone), which produce reactive oxygen species (ROS). These reactive metabolites and ROS can damage both pre- and post-synaptic membranes in the brain, ultimately resulting in neuronal cell damage [24]. Synaptosomes were incubated with 6-OHDA (150 μM) for 1 h.

#### 3.2.5. MTT Assay to Assess Synaptosomal Viability

Synaptosomes’ viability was assessed by an MTT test described by Mungarro-Menchaca et al. [34], with minor modifications. After 1 h of incubation with the substances and toxic agent, synaptosomes (1 mL) were centrifuged on a microcentrifuge for 1 min at 15,000× *g*. The pellet was mixed gently with 600 µL buffer B + glucose, and the supernatant where 6-OHDA was discarded to prevent the oxidation of MTT was centrifuged again at 15,000× *g* for 1 min. After the second wash, 600 µL buffer B + glucose was added to the pellet. An amount of 60 μL of MTT solution was added to the washed synaptosomes. The plates were incubated with the MTT solution at 37 °C for 10 min. After incubation, the samples were centrifuged at 15,000× *g* for 2 min. The excess liquid was removed, and a 300 µL DMSO solution was used to dissolve the formed formazan crystals. After dissolution, the amount of formazan was measured spectrophotometrically at λ = 580 nm.

#### 3.2.6. Determination of Reduced Glutathione (GSH) in Isolated Brain Synaptosomes

The reduced GSH in isolated brain synaptosomes was measured using the protocol described in the literature [35], with minor modifications. After the precipitation of the proteins with trichloroacetic acid, the thiol groups in the supernatant were determined by DTNB, which produced a yellow-colored compound that absorbs light at λ = 412 nm. After incubation, synaptosomes (500 µL) were centrifuged at 4000× *g* for 3 min. The supernatant was removed, and the pellet was taken for GSH determination. It was treated with 500 µL 5% trichloroacetic acid, left for 10 min on ice, and then centrifuged at 8000× *g* for 10 min (20 °C). The supernatant was taken for GSH determination and frozen at −20 °C. Immediately before measurement, the samples were neutralized with 15 µL 5 N NaOH.

#### 3.2.7. Tert-Butyl Hydroperoxide-Induced Oxidative Stress

Isolated brain mitochondria were incubated with 75 μM tert-butyl hydroperoxide (t-BuOOH) for 1 h according to the procedure described by Karlsson et al. [26].

#### 3.2.8. Determination of Malondialdehyde (MDA) Production in Brain Mitochondria

The malondialdehyde (MDA) production in brain mitochondria was established according to the Shirani et al. procedure, with small modifications [36]. Briefly, 0.3 mL of 0.2% thiobarbituric acid and 0.25 mL of sulfuric acid (0.05 M) were added to the mitochondria, and the mixture was boiled for 30 min. Then, the tubes were placed on ice, and 0.4 mL of n-butanol was added to each. The tubes were centrifuged at 3500× *g* for 10 min. The amount of MDA was determined spectrophotometrically at 532 nm.

#### 3.2.9. Determination of GSH Level in Brain Mitochondria

The GSH level in brain mitochondria was established according to the procedure described by Shirani et al., with minor modifications [36]. Briefly, after the mitochondria were incubated with the substances and t-BuOOH, the reaction was stopped with 500 µL 5% trichloroacetic acid, and each sample was homogenized with the acid and left on ice for 10 min. After the centrifugation of the homogenate at 6000× *g* for 10 min, a 500 µL 0.04% solution of DTNB was added to the supernatant to give it a yellow color, with the determination being spectrophotometric at 412 nm.

#### 3.2.10. Isolation of Brain Microsomes

The brain microsomes were isolated by following the protocol described by Ravindranath et al. [37], with minor modifications. The rat brains were homogenized in nine volume parts of Tris buffer containing dithiothreitol, phenylmethylsulfonyl fluoride, EDTA, KCl, and glycerol (pH 7.4). The resulting homogenate (20 mL) was centrifuged twice at 17,000× *g* for 30 min. The supernatants from the two centrifugations were pooled and centrifuged twice at 100,000× *g* for 1 h. The pellet was frozen in 0.1 M Tris buffer.

#### 3.2.11. Iron/Ascorbate-Induced Lipid Peroxidation (LPO)

Non-enzyme-induced lipid peroxidation was induced with 20 μM ferrous sulfate solution and 0.5 mM ascorbic acid solution for 1 h. Mansuy et al. described the protocol [38].

#### 3.2.12. Determination of MDA in Brain Microsomes

The protocol for measuring the MDA levels in brain microsomes was described by Mansuy et al. [38]. After ending the incubation of the microsomes with the substances and toxic agent, the reaction was stopped with 0.5 mL of 20% trichloroacetic acid followed by 0.5 mL of 0.67% thiobarbituric acid. The mixture was boiled for 35 min. Then, the tubes were placed on ice for 10 min. The tubes were centrifuged at 6000× *g* for 10 min. The ongoing reactions were associated with forming a colored complex between the malondialdehyde formed and thiobarbituric acid. The determination of MDA was spectrophotometric at 535 nm. A molar extinction coefficient of 1.56 × 10^5^ M^−1^ cm^−1^ was used for the calculation.

#### 3.2.13. Statistical Methods

The results of the experiments performed on isolated brain synaptosomes, mitochondria, and microsomes were statistically analyzed using the non-parametric Mann–Whitney method at significance levels *p* < 0.05, *p* < 0.01, and *p* < 0.001 with the “MEDCALC” program. The statistical processing of hMAO-A and hMAO-B activity results was performed using GraphPad Prism 5.0 software. Statistical significance in comparing means was determined by the Student’s test and Tukey–Kramer Multiple Comparison test for paired and group data at a statistical significance of *p* < 0.05.

### 3.3. A UHPLC-HRMS Method for Identification and Simultaneous Quantification of Flavonoids and Saponins Present in the Aerial Parts of C. foliosum *Asch.*

#### 3.3.1. Apparatus

The UHPLC-HRMS analysis was performed using a Thermo Scientific Dionex Ultimate 3000 RSLC (Germering, Germany) consisting of a 6-channel degasser SRD-3600, high-pressure gradient pump HPG-3400RS, autosampler WPS-3000TRS, and column compartment TCC-3000RS coupled to a Thermo Scientific Q Exactive Plus (Bremen, Germany) mass spectrometer.

#### 3.3.2. Plant Material

The aerial parts of *Chenopodium foliosum* Asch. were collected from Beglica, Western Rhodopes, Bulgaria, in June 2020. Z. Kokanova-Nedialkova identified the plant, and a voucher specimen (No. SOM-178269) from the plant population was deposited at the National Herbarium of the Institute of Biodiversity and Ecosystem Research (IBER) at the Bulgarian Academy of Sciences (BAS), Sofia, Bulgaria.

#### 3.3.3. Extraction and Sample Preparation

The aerial parts of *C. foliosum* Asch. were dried in the shade, and the powdered plant material (100 mg) was extracted with 70 vol. % EtOH (30 mL) by ultrasonic-assisted extraction for 30 min. Then, it was diluted to 50 mL with the same solvent. The resulting solution was filtered, and the first 10 mL was removed. An aliquot (10 mL) of this solution was evaporated to dryness, then dissolved in water and further purified by solid-phase extraction over Phenomenex (Torrance, CA, USA) Strata C18-E (55 μm, 70 A, 200 mg, 3 mL) cartridge. The sorbent was first washed with H_2_O, then eluted with 70 vol. % EtOH (12 × 500 μL) in a 10.0 mL volumetric flask and diluted to the nominal volume with the same solvent (solution A). Subsequently, 1 mL of solution A was diluted to 10 mL 70 vol. % EtOH (solution B). The latter solution was used for the qualitative and quantitative analysis of flavonoids and saponins by UHPLC–ESI-MS/MS.

#### 3.3.4. UHPLC Chromatographic Conditions

UHPLC separations were performed on a Kromasil Eternity XT C18 column (AkzoNobel, Sweden) (2.1 × 100 mm, 1.8 μm) equipped with precolumn SecurityGuard ULTRA UHPLC EVO C18 (Phenomenex, USA) at 40 °C. Each chromatographic run was carried out with a binary mobile phase consisting of water containing 0.1% (*v*/*v*) formic acid (A) and acetonitrile also with 0.1% (*v*/*v*) formic acid (B). A gradient program was used as follows: 0–0.5 min, 13% B; 0.5–5 min, 13–15% B; 5–8 min, 15–18% B; 8–16 min, 18–25% B; 16–20 min, 25% B; 20–26 min, 25–38% B; 26–27 min, 38–95% B; and 27–29.5 min, 95% B. The flow rate was 0.3 mL·min^−1,^ and the sample injection volume was 2 μL.

#### 3.3.5. High-Resolution Electrospray Ionization Mass Spectrometry (HRESIMS) Conditions

The operating conditions for the HESI source used in a negative ionization mode were as follows: −2.5 kV spray voltage, 320 °C capillary and probe heater temperature, sheath gas flow rate 38 a.u., auxiliary gas flow 12 a.u. (a.u. refer to arbitrary values set by the Exactive Tune 2.8 SP1 build 2806 software) and S-Lens RF level 50.00. Nitrogen was used for sample nebulization and collision gas in the HCD cell. The full MS mode was used as an MS experiment where the resolution, AGC target, max. IT, and mass range were 70,000 (at *m/z* 200), 3 × 10^6^, 200 ms, and *m/z* 300–1500, respectively. The deprotonated molecules [M−H]^−^ at *m/z* 463.0871 (for hyperoside) and *m/z* 809.3954 (for Chfs01), with a 10.0 ppm isolation window, were used as quantifiers. The data were acquired and processed with Thermo Fisher Scientific Xcalibur ver. 4.1 and FreeStyle ver. 1.8 SP2 QF1. The operating conditions for the HESI source used in a positive ionization mode were previously described in detail elsewhere [32].

#### 3.3.6. Method Validation

Flavonoids and saponins were quantified using the external standard method. The amount of 28 detected compounds was calculated relative to external hyperoside and saponin Chfs01 standards. Each external standard (about 5 mg) was dissolved in 50 mL 70 vol. % EtOH (primary solutions). The stock standard solution of the external standards was prepared by combining the aliquots (1 mL) of each primary solution and dilution to 20 mL with 70 vol. % EtOH. The working standard solutions of appropriate concentration were prepared by diluting the stock standard solution with 70 vol. % EtOH. External standard calibrations were established on six data points covering the concentration range of 16.41–525.00 ng/mL for hyperoside, and 17.97–575.00 ng/mL for **Chfs01**. The procedure was according to the ICH guidelines [29], and the validation parameters were previously described by Kokanova-Nedialkova and Nedialkov [28].

## 4. Conclusions

Fifteen flavonoids and saponins from *C. foliosum* Asch. and *C. bonus*-*henricus* L. were tested for their inhibitory activity on hMAO-A and hMAO-B. Five compounds (**11**–**15**) (1 μM) showed weak inhibition of hMAO-A but exhibited good inhibitory activity (30–35%) of hMAO-B enzyme, compared to the positive control, selegiline (55%). These active compounds were also tested in various brain subcellular fractions, including rat synaptosomes, mitochondria, and microsomes. At 50 μM, they demonstrated significant neuroprotective and antioxidant properties across induced oxidative stress models (6-OHDA, t-BuOOH, and Fe^2+/^AA-induced lipid peroxidation). In a 6-hydroxydopamine-induced neurotoxicity model on synaptosomes, glycosides of spinacetin and gomphrenol showed the strongest neuroprotective effects, preserving 60% synaptosome viability and 70% GSH levels, compared to silybin (70%). In a tert-butyl hydroperoxide-induced mitochondrial oxidative stress model, gomphrenol glycosides showed the highest activity, preserving 70% GSH levels and reducing MDA production by 35–36%, compared to silybin (80% GSH, 44% MDA). In a model of non-enzyme-induced lipid peroxidation on isolated rat brain microsome, glycosides of spinacetin and gomphrenol reduced MDA production by 43–48%, close to silybin’s 51%. Neuroprotection likely occurs via MAO-B inhibition, ROS scavenging, cell membrane stabilization (via reduced MDA), and free radical neutralization (by maintaining GSH levels).

A novel UHPLC-HRMS method was developed to identify and quantify 28 compounds (20 flavonoids, 8 saponins) in the *C. foliosum* EtOH extract. Two new natural compounds, 30-normedicagenic acid-HexA-Hex-TA (**22f**) and medicagenic acid-HexA-Hex-TA (**25f**), were discovered. Notably, gomphrenol glycosides (**14**, **15**) and 30-normedicagenic acid glycosides (**12**, **13**) accounted for 37.10% and 43.70% of the total flavonoid and saponin content, respectively. These findings highlight *C. foliosum* as a promising source of neuroprotective and antioxidant compounds, supporting its use in Bulgarian folk medicine. Future research could explore the synergistic effects of these biologically active substances.

## Figures and Tables

**Table 1:** Linearity of calibration curve for the hyperoside and saponin **Chfs01**.

External Standard	Linear Range (ng/mL)	Regression Equation	R^2^	LOD (ng/mL)	LOQ (ng/mL)
Hyperoside	16.41–525.00	Y = 83,212*X − 503,210	0.9993	0.28	0.86
Saponin **Chfs01**	17.97–575.00	Y = 31,732*X − 3951.1	0.9994	0.48	1.44

**Table 2:** Accuracy of the UHPLC-HRMS method.

External Standard	Added (ng/mL)	Found * (ng/mL)	Recovery * (%)	RSD (%)
Hyperoside	50.2	49.43 ± 0.19	98.46 ± 0.37	0.38
	100.4	99.46 ± 1.22	99.06 ± 1.21	1.22
	150.6	150.71 ± 1.00	100.07 ± 0.66	0.66
Saponin **Chfs01**	50.8	50.16 ± 0.39	98.73 ± 0.76	0.77
	101.6	103.68 ± 0.61	102.05 ± 0.60	0.58
	152.4	154.97 ± 0.60	101.69 ± 0.39	0.38

* Values are the mean ± SD (*n* = 3).

**Table 3:** Evaluation of intra-day (repeatability) and inter-day (intermediate precision) precision of the UHPLC-HRMS method applied on hyperoside and saponin **Chfs01**.

Precision Type	RT ± SD (min)	RSD (%)	Recovery ± SD (%)	RSD (%)
Hyperoside				
Intra-day	7.78 ± 0.01	0.07	98.94 ± 0.49	0.50
Inter-day	7.78 ± 0.001	0.01	98.21 ± 0.17	0.18
Saponin **Chfs01**				
Intra-day	19.71 ± 0.01	0.06	97.67 ± 0.62	0.64
Inter-day	19.71 ± 0.001	0.003	97.11 ± 0.42	0.44

**Table 4:** The quantity of detected flavonoids and saponins in the aerial parts of *C. foliosum* Asch.

№	Compound	RT (min)	Quantifier Ion	Ion Mode	µg/g D.W. ± SD	Calc. ^1^
**1f**	6-methoxykaempferol-Glu-Glu-Rham	5.12	785.2167	[M−H]^−^	24.02 ± 0.91	H
**2f**	Patuletin-3-*O*-β-Glu(1→6)-β-Glu	6.00	655.1530	[M−H]^−^	66.70 ± 0.50	H
**3+4f**	6-methoxykaempferol-3-*O*-[β-Api (1→2)]-β-Glu (1→6)-β-Glu	6.61	771.2004	[M−H]^−^	150.17 ± 2.00	H
**5f**	Spinacetin-3-*O*-[β-Api (1→2)]-β-Glu (1→6)-β-Glu	7.18	801.2115	[M−H]^−^	36.54 ± 1.26	H
**6f**	6-methoxykaempferol-3-*O*-β-Glu(1→6)-β-Glu	8.31	639.1576	[M−H]^−^	326.65 ± 0.36	H
**7f**	Spinacetin-3-*O*-β-Glu (1→6)-β-Glu	9.08	669.1828	[M−H]^−^	in traces	H
**8f**	Gomphrenol-3-*O*-α-L-Rham (1→2)[β-D-Glu(1→6)]-β-D-Glu	9.18	783.2003	[M−H]^−^	345.09 ± 0.82	H
**9f**	FLB-Glu-Glu-Rham	9.40	813.2116	[M−H]^−^	40.04 ± 0.32	H
**10f**	FLA-Glu-Glu	9.55	653.1374	[M−H]^−^	16.66 ± 0.30	H
**11f**	Gomphrenol-Glu-Glu-Api	10.29	769.1839	[M−H]^−^	722.80 ± 1.59	H
**12f**	FLB-Glu-Glu-Api	10.73	799.1957	[M−H]^−^	91.55 ± 0.27	H
**13f**	Patuletin-3-*O*-(5‴-O-E-FA)-β-D-Api (1→2) [β-D-Glu (1→6)]-β-D-Glu	11.44	963.2435	[M−H]^−^	40.82 ± 0.89	H
14f *	Gomphrenol-3-*O*-β-Glu(1→6)-β-Glu (Compound **14**)	11.95	637.1414	[M−H]^−^	574.16 ± 5.06	H
**15f**	FLB-Glu-Glu	12.54	667.1531	[M−H]^−^	94.41 ± 0.14	H
**16f**	Spinacetin-3-*O*-(5‴-*O*-E-FA)-β-D-Api(1→2)[β-D-Glu (1→6)]-β-D-Glu	12.67	977.2589	[M−H]^−^	74.83 ± 1.14	H
**17f**	6-methoxykaempferol-3-*O*-(5‴-*O*-E-FA)-β-D-Api(1→2)[β-D-Glu (1→6)]-β-D-Glu	13.08	947.2476	[M−H]^−^	305.41 ± 0.84	H
**18f**	FLA-Glu-Glu-Api-FA	14.20	961.2275	[M−H]^−^	51.02 ± 0.16	H
**19f**	FLB-Glu-Glu-Api-FA	15.65	975.2431	[M−H]^−^	127.45 ± 2.96	H
**20f** *	Gomphrenol-3-*O*-(5‴-*O*-E-FA)-β-D-Api(1→2)[β-D-Glu (1→6)]-β-D-Glu (Compound **15**)	15.98	945.2311	[M−H]^−^	909.03 ± 10.68	H
**21f**	3-*O*-β-GluA-30-normedicagenic acid-28-*O*-β-Glu	19.29	823.3766	[M−H]^−^	1408.06 ± 2.37	**Chfs01**
**22f**	30-normedicagenic acid-HexA-Hex-TA	19.56	955.3828	[M−H]^−^	496.98 ± 1.13	**Chfs01**
**23f** *	3-*O*-β-Glu-30-normedicagenic acid-28-*O*-β-Glu (Compound **12**)	19.82	809.3972	[M−H]^−^	1735.11 ± 11.75	**Chfs01**
**24f**	Medicagenic acid-HexA-Hex	21.92	839.4079	[M−H]^−^	366.67 ± 1.56	**Chfs01**
**25f**	Medicagenic acid-HexA-Hex-TA	22.14	971.4143	[M−H]^−^	171.53 ± 2.84	**Chfs01**
**26f**	Medicagenic acid-Hex-Hex	22.39	825.4295	[M−H]^−^	105.46 ± 0.31	**Chfs01**
**27f**	30-normedicagenic acid-HexA	25.74	661.3237	[M−H]^−^	396.61 ± 1.48	**Chfs01**
**28f** *	3-*O*-β-Glu-30-normedicagenic acid (Compound **13**)	26.00	647.3442	[M−H]^−^	551.17 ± 5.83	**Chfs01**

FLA, 3,5,3′,4′-tetrahydroxy-6,7-methylenedioxyflavone; FLB, 3,5,4′-trihydroxy-3′-methoxy-6,7-methylenedioxyflavone; Glu, glucose; Api, apiose; Rham, rhamnose; Hex, hexose; HexA, hexauronic acid; FA, ferulic acid; and TA, tartaric acid. ^1^ The quantity of the metabolites was calculated as hyperoside (H) and 3-*O*-β-Glu-30-normedicagenic acid-28-*O*-β-Glu (**Chfs01**). * The compounds were tested for hMAO-A/B and neuroprotective activity in this study.

## Data Availability

Data are contained within the article and in the Supplementary Material.

## Supplementary Materials

The following supporting information can be downloaded at https://www.mdpi.com/article/10.3390/molecules30051061/s1: Table S1: UHPLC-HRMS characterization of flavonoids and saponins from EtOH extract of the aerial parts of *C. foliosum* Asch. in the positive and negative ion modes.
