# Supplementary material for: Flavonoids and Saponins from Two Chenopodium Species (C. foliosum Asch. and C. bonus-henricus L.)—Preliminary Evaluation for hMAO-A/B, Neuroprotective Activity, and Validated UHPLC-HRMS Quantification of Ethanolic Extract from C. foliosum
Source: Molecules. 2025 Feb 26;30(5):1061. doi: 10.3390/molecules30051061 (PMC11901915; doi:10.3390/molecules30051061)
Supplement: Supplementary file 1 [file molecules-30-01061-s001.zip › molecules-3475746-supplementary.pdf]

Table S1. UHPLC-HRMS characterization of flavonoids and saponins from EtOH extract of the aerial parts of *Chenopodium foliosum* Asch.

**№**	**$t_{R\text{-}extract}$[1]**	**Identification**	**Ion type**	**Found *m/z***	**Calculated *m/z***	**Composition (*δ* ppm)**	**Fragment ions (*m/z*)**
**1f**	5.09	6-methoxykaempferol-Glu-Glu-Rham	$[M+H]^+$	787.2283	787.2291	$C_{34}H_{43}O_{21}$ (2.0)	641.1716 $[M+H-Rham]^+$<br>479.1190 $[M+H-Rham-Glu]^+$<br>317.0654 $[M+H-Rham-Glu-Glu]^+$
**2f[2]**	5.99	Patuletin-3-O-*β*-Glu(1→6)-*β*-Glu	$[M+H]^+$	657.1672	657.1661	$C_{28}H_{33}O_{18}$ (1.58)	495.1127 $[M+H-Glu]^+$<br>333.0600 $[M+H-Glu-Glu]^+$
**3f[2]**	6.59	6-methoxykaempferol-3-O-[*β*-Api (1→2)]-*β*-Glu (1→6)-*β*-Glu	$[M+H]^+$	773.2141	773.2135	$C_{33}H_{41}O_{21}$ (0.81)	641.1697 $[M+H-Api]^+$<br>479.1177 $[M+H-Api-Glu]^+$<br>317.0652 $[M+H-Api-Glu-Glu]^+$
**4f**	6.59	Isorhamnetin-Glu-Glu-Api	$[M+H]^+$	773.2141	773.2135	$C_{33}H_{41}O_{21}$ (0.81)	641.1697 $[M+H-Api]^+$<br>479.1177 $[M+H-Api-Glu]^+$<br>317.0652 $[M+H-Api-Glu-Glu]^+$
**5f[2]**	7.17	Spinacetin-3-O-[*β*-Api (1→2)]-*β*-Glu (1→6)-*β*-Glu	$[M+H]^+$	803.2251	803.2240	$C_{34}H_{43}O_{22}$ (1.31)	671.1805 $[M+H-Api]^+$<br>509.1274 $[M+H-Api-Glu]^+$<br>347.0757 $[M+H-Api-Glu-Glu]^+$
**6f[2]**	8.30	6-methoxykaempferol-3-O-*β*-Glu(1→6)-*β*-Glu	$[M+H]^+$	641.1720	641.1712	$C_{28}H_{33}O_{17}$ (1.21)	479.1179 $[M+H-Glu]^+$<br>317.0652 $[M+H-Glu-Glu]^+$
**7f[2]**	9.08	Spinacetin-3-O-*β*-Glu(1→6)-*β*-Glu	$[M+H]^+$	671.1826	671.1818	$C_{29}H_{35}O_{18}$ (1.24)	509.1287 $[M+H-Glu]^+$<br>347.0757 $[M+H-Glu-Glu]^+$
**8f[2]**	9.17	Gomphrenol-3-O-*α*-L-Rham (1→2)[*β*-D-Glu(1→6)]-*β*-D-Glu	$[M+H]^+$	785.2142	785.2135	$C_{34}H_{41}O_{21}$ (0.87)	639.1550 $[M+H-Rham]^+$<br>477.1025 $[M+H-Rham-Glu]^+$<br>315.0496 $[M+H-Rham-Glu-Glu]^+$
**9f**	9.38	FLB-Glu-Glu-Rham	$[M+H]^+$	815.2253	815.2240	$C_{35}H_{43}O_{22}$ (1.58)	669.1646 $[M+H-Rham]^+$<br>507.1131 $[M+H-Rham-Glu]^+$<br>345.0600 $[M+H-Rham-Glu-Glu]^+$
**10f**	9.51	FLA-Glu-Glu	$[M+H]^+$	655.1517	655.1505	$C_{28}H_{31}O_{18}$ (1.8)	493.0973 $[M+H-Glu]^+$<br>331.0445 $[M+H-Glu-Glu]^+$
**11f**	10.28	Gomphrenol-Glu-Glu-Api	$[M+H]^+$	771.1978	771.1978	$C_{33}H_{39}O_{21}$ (-0.03)	639.1551 $[M+H-Api]^+$<br>477.1026 $[M+H-Api-Glu]^+$<br>315.0495 $[M+H-Api-Glc-Glc]^+$
**12f**	10.71	FLB-Glu-Glu-Api	$[M+H]^+$	801.2094	801.2084	$C_{34}H_{41}O_{22}$ (1.26)	669.1653 $[M+H-Api]^+$<br>507.1140 $[M+H-Api-Glu]^+$<br>345.0600 $[M+H-Api-Glu-Glu]^+$

**13f**[2]	11.44	Patuletin-3-O-(5‴-O-E-FA)-*β*-D-Api (1→2) [*β*-D-Glu (1→6)]-*β*-D-Glu	[M+H]$^+$	965.2574	965.2557	$C_{43}H_{49}O_{25}$ (1.77)	803.2039 [M+H−Glu]$^+$<br>657.1653 [M+H−FA−Api]$^+$<br>495.1130 [M+H−FA−Api−Glu]$^+$<br>333.0600 [M+H−FA−Api−Glu−Glu]$^+$<br>177.0546 Feruloyl$^+$
**14f**[2]	11.94	Gomphrenol-3-O-*β*-Glu(1→6)-*β*-Glu	[M+H]$^+$	639.1563	639.1556	$C_{28}H_{31}O_{17}$ (1.15)	477.1024 [M+H−Glu]$^+$<br>315.0496 [M+H−Glu−Glu]$^+$
**15f**	12.52	FLB-Glu-Glu	[M+H]$^+$	669.1672	669.1661	$C_{29}H_{33}O_{18}$ (1.64)	507.1132 [M+H−Glu]$^+$<br>345.0600 [M+H−Glu−Glu]$^+$
**16f**[2]	12.63	Spinacetin-3-O-(5‴-O-*E*-FA)-*β*-D-Api(1→2)[*β*-D-Glu (1→6)]-*β*-D-Glu	[M+H]$^+$	979.2731	979.2714	$C_{44}H_{51}O_{25}$ (1.77)	817.2166 [M+H−Glu]$^+$<br>671.1794 [M+H−FA−Api]$^+$<br>509.1289 [M+H−FA−Api−Glu]$^+$<br>347.0756 [M+H−FA−Api−Glu−Glu]$^+$<br>177.0545 Feruloyl$^+$
**17f**[2]	13.06	6-methoxykaempferol-3-O-(5‴-O-E-FA)-*β*-D-Api(1→2)[*β*-D-Glu (1→6)]-*β*-D-Glu	[M+H]$^+$	949.2620	949.2608	$C_{43}H_{49}O_{24}$ (1.26)	787.1981 [M+H−Glu]$^+$<br>641.1721 [M+H−FA−Api]$^+$<br>479.1182 [M+H−FA−Api−Glu]$^+$<br>317.0653 [M+H−FA−Api−Glu−Glu]$^+$<br>177.0546 Feruloyl$^+$
**18f**	14.19	FLA-Glu-Glu-Api-FA	[M+H]$^+$	963.2418	963.2401	$C_{43}H_{47}O_{25}$ (1.73)	801.1859 [M+H−Glu]$^+$<br>655.1516 [M+H−FA−Api]$^+$<br>493.0977 [M+H−FA−Api−Glu]$^+$<br>331.0445 [M+H−FA−Api−Glu−Hex]$^+$<br>177.0545 Feruloyl$^+$
**19f**	15.63	FLB-Glu-Glu-Api-FA	[M+H]$^+$	977.2574	977.2557	$C_{44}H_{49}O_{25}$ (1.75)	815.2059 [M+H−Glu]$^+$<br>669.1655 [M+H−FA−Api]$^+$<br>507.1133 [M+H−FA−Api−Glu]$^+$<br>345.0602 [M+H−FA−Api−Glu−Glu]$^+$<br>177.0546 Feruloyl$^+$
**20f**[2]	15.97	Gomphrenol-3-*O*-(5'''-*O*-E-FA)-*β*-D-Api(1→2)[*β*-D-Glu (1→6)]-*β*-D-Glu	[M+H]$^+$	947.2465	947.2452	$C_{43}H_{47}O_{24}$ (1.35)	785.1887 [M+H−Glu]$^+$<br>639.1553 [M+H−FA−Api]$^+$<br>477.1026 [M+H−FA−Api−Glu]$^+$<br>315.0497 [M+H−FA−Api−Glu−Glu]$^+$<br>177.0547 Feruloyl$^+$
**21f**[2]	19.24	3-O-β-GluA-30-normedicagenic acid-28-O-β-Glu	[M−H]$^-$	823.3767	823.3747	$C_{41}H_{59}O_{17}$ (2.4)	485.2908 [M−H−GluA−Glu]$^-$
**22f**	19.53	30-normedicagenic acid- HexA-Hex-TA	[M−H]$^-$	955.3835	955.3806	$C_{45}H_{63}O_{22}$ (3.13)	823.3763 [M−H−TA]$^-$<br>647.3480 [M−H−TA−HexA]$^-$<br>485.2900 [M−H−TA−HexA−Hex]$^-$<br>131.0337 Tartaloyl$^-$

**23f**[2]	19.81	3-O-β-Glu-30-normedicagenic acid-28-O-β-Glu	[M−H]⁻	809.3978	809.3954	$C_{41}H_{61}O_{16}$ (2.99)	647.3413 [M−H−Glu]⁻<br>423. 2906 [M−H−Glu−Glu−H_2O−CO_2]⁻
**24f**	21.88	Medicagenic acid-HexA-Hex	[M−H]⁻	839.4090	839.4060	$C_{42}H_{63}O_{17}$ (3.6)	663.3695 [M−H−HexA]⁻<br>501.3247 [M−H−HexA−Hex]⁻
**25f**	22.12	Medicagenic acid-HexA-Hex-TA	[M−H]⁻	971.4156	971.4119	$C_{46}H_{67}O_{22}$ (3.85)	839.4084 [M−H−TA]⁻<br>663.3749 [M−H−TA−HexA]⁻<br>501.3250 [M−H−TA−HexA-Hex]⁻<br>131.0337 Tartaloyl⁻
**26f**	22.36	Medicagenic acid-Hex-Hex	[M−H]⁻	825.4304	825.4267	C_{42} H_{65} O_{16} (4.49)	439.3207 [M−H−Hex−Hex−H_2O−CO_2]⁻
**27f**	25.71	30-normedicagenic acid-HexA	[M−H]⁻	661.3244	661.3219	$C_{35}H_{49}O_{12}$ (3.85)	485.2898 [M−H−HexA]⁻
**28f**[2]	25.97	3-O-β-Glu-30-normedicagenic acid	[M−H]⁻	647.3449	647.3426	$C_{35}H_{51}O_{11}$ (3.49)	423.2906 [M−H−Glu−H_2O−CO_2]⁻

[1]Retention times (min) of compounds **1f-28f** in the EtOH extract;

[2]Flavonoids and saponins compared with a reference compound;

FLA = 3,5,3',4'-tetrahydroxy-6,7-methylenedioxyflavone; FLB = 3,5,4'-trihydroxy-3'-methoxy-6,7-methylenedioxyflavone; Glu = glucose; Api = apiose; Rham = rhamnose; Hex = hexose; HexA = hexauronic acid; FA = ferulic acid; TA = tartaric acid.
